# Current and emerging opportunities for molecular simulations in structure-based drug design

**DOI:** 10.1039/c3cp54164a

**Published:** 2014-01-28

**Authors:** Julien Michel

**Affiliations:** a EaStCHEM School of Chemistry , Joseph Black Building , The King's Buildings , Edinburgh , EH9 3JJ , UK . Email: mail@julienmichel.net

## Abstract

An overview of the current capabilities and limitations of molecular simulation of biomolecular complexes in the context of computer-aided drug design is provided. Steady improvements in computer hardware coupled with more refined representations of energetics are leading to a new appreciation of the driving forces of molecular recognition. Molecular simulations are poised to more frequently guide the interpretation of biophysical measurements of biomolecular complexes. Ligand design strategies emerge from detailed analyses of computed structural ensembles. The feasibility of routine applications to ligand optimization problems hinges upon successful extensive large scale validation studies and the development of protocols to intelligently automate computations.

## Abstract 2

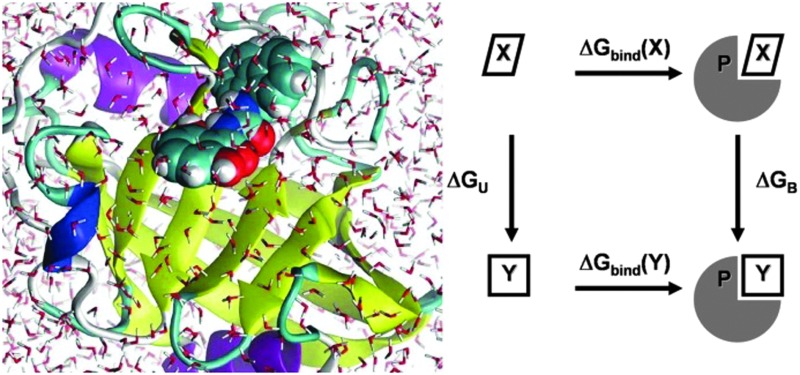
Opportunities are reviewed for state-of-the-art molecular simulations to progress the understanding of the molecular driving forces of protein–ligand association, assist interpretation of biophysical measurements, and contribute to structure-based drug design efforts.

## 1. Introduction

The field of computer-aided drug design emerged over 40 years ago; early seminal work can be traced to Corwin Hansh's efforts to derive structure–activity relationships, or Graham Richard's influential book Quantum Pharmacology. Today computer modelling is frequently relied upon in some ways in the pharmaceutical industry and academic laboratories to assist early stage drug discovery activities.^1^ Ubiquitous examples include small molecule/biomolecule database searches by chemical/sequence similarity, homology modelling, conformational searches and sketching of putative ligands in target binding sites. Early enthusiasm in the 1980s for the field faltered when it became apparent that more sophisticated applications, such as virtual screens for finding or optimizing hits into suitable clinical candidates, were finding mixed success.^2^ Currently a typical virtual screen by docking exercise entails processing a database of *ca.* 10^5^–10^7^ small molecules, followed up by extensive post-filtering that leads to *in vitro* assays for 10^1^–10^3^ compounds. Success is usually declared when perhaps over 10% of the tested compounds show some measurable bioactivity, typically an IC_50_ or *K*_d_ in the μM range. Although it is unsatisfactory that about 90% of the time activity predictions are not corroborated by experiments, a pragmatic view is that the outcome is acceptable if it leads to the identification of a few novel scaffolds. Follow up work is almost invariably necessary as initial hits rarely exhibit desirable potency, selectivity and ADME properties to be progressed to *in vivo* disease models. A typical early focus is on improving the potency of a hit compound by 3–4 orders of magnitude to produce a lead compound. Later stage considerations involve further modifications of a lead molecule that maintain potency whilst improving binding selectivity, solubility, cell permeability, and metabolic stability among others. The process is usually pursued by iterative synthesis and assaying of analogues of a parent structure, which requires significant time and resource commitments. A long standing goal of computational chemistry has been to replace most of this iterative process with much cheaper computational methods.

The relative potencies of a typical hit (low-mid μM *K*_d_) and lead compound (low nM *K*_d_) correspond to a change in the Gibbs free energy of binding of *ca.* 4–6 kcal mol^–1^. Simple statistical analyses of affinity changes observed in multiple past hit-to-lead campaigns suggest that achieving significant time and cost savings with computer modelling requires consistent predictions of relative binding free energies to within *ca.* 2 kcal mol^–1^ or better.^3^ Achieving this objective has proven much harder than it was anticipated in the mid-1980s when it became possible to readily obtain the crystallographic structure of protein–ligands complexes. Tantalizing as they are, three dimensional structures of biomolecular complexes only capture some aspects of molecular recognition. “Details” such as solvent rearrangements and fluctuations, induced fit, changes in ligand–receptor conformational entropy, treatment of electrostatics–polarization, matter greatly.^4^ Today, it is widely assumed that achieving sufficient accuracy in binding energy predictions to enable routine ligand optimization with computational methods will require detailed explicit solvent equilibrium molecular simulations of biomolecular complexes.

Biomolecular simulations have progressed greatly since the 1977 landmark 10 ps vacuum molecular dynamics study of the protein BPTI by McCammon, Gelin and Karplus.^5^ Protein folding studies now reach millisecond timescales on dedicated supercomputers such as the Anton machine produced by the DE Shaw lab,^6^ whereas large distributed computing projects such as Folding@Home from the Pande lab can rapidly produce even higher aggregated sampling time.^7^ Although these high-end systems are only available to a chosen few, advances in GPU-computing have in parallel dramatically improved prospects for routine use of simulations in drug discovery.^8^ Although these trends strongly suggest that the scope of routine biomolecular simulations will continue expanding its reach to larger time and length scales, it is still unclear whether the current typical potential energy functions are sufficiently accurate and transferable for reliable routine applications to structure-based drug design problems. In other words, successful molecular simulation “recipes” remain to be validated or even discovered, and widely taken up in routine industrial drug design workflows. In addition, many complex biomolecular recognition processes of direct relevance to drug design will remain only partially accessible to the most advanced simulation protocols and supercomputers for some time (*e.g.* cell surface receptor activation, DNA transcription), thus it is important to appreciate what objectives can be productively pursued with molecular simulations today and in the near future.

**Fig. 1:**
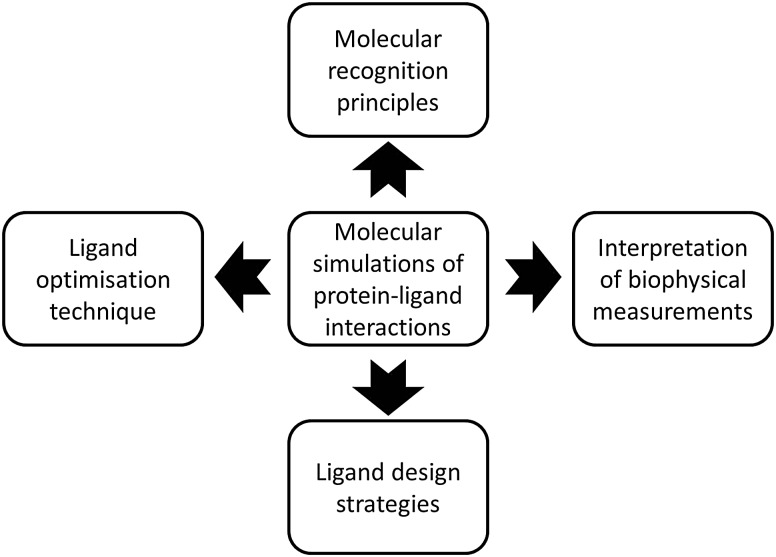
Four strategic applications of molecular simulations of protein–ligand interactions to relevant structure-based drug design problems.

This perspective will discuss some of the current opportunities molecular simulations have to contribute to structure-based drug design efforts. Fig. 1 summarizes the four major topics covered by this perspective. For clarity each topic has been further divided into different sub-sections. The focus on small molecule–protein complexes reflects my own biases and current interests, other topics in biomolecular simulation are certainly important. Emphasis is put on concepts and challenges, several recent excellent reviews can be consulted elsewhere for technical details on specific methodologies.^3,4,9–15^ The field is vast and not all excellent work from colleagues could be possibly covered, it is hoped that the issues discussed here will stimulate further research.

## 2. Opportunities to explore principles of molecular recognition

### 2.1 Non-additivity of protein–ligand interactions

Rational attempts to sequentially optimize the affinity of a hit compound are frequently thwarted by the non-additivity of protein–ligand interactions. In other words, when two structural modifications, that each individually improves affinity, are introduced on distinctive parts of a ligand, the resulting analogue may not yield the expected affinity improvement.^16^ This outcome is often difficult to anticipate for computational methods that rely on analyses of a static protein–ligand structure.^17^ When non-additive effects are observed, conformational changes and changes in entropy are frequently invoked after the facts, but convincing evidence or structural insights into the origin of non-additivity often remains elusive. Molecular simulations of protein–ligand complexes account in principle for a broad range of plausible sources of non-additivity. In particular, detailed analyses can be currently attempted to characterize the contribution of solvent, ligand or protein molecules to the free energies of binding. Breaking down binding free energies into components presents its own pitfalls because the free energy components are not state-functions, *i.e.* alternative ways to partition the binding free energy into contributions from different groups are equally valid. Nonetheless, it is reasonable to expect that partitioning schemes justified on physical-chemistry grounds can provide insights into the molecular driving forces of protein–ligand association.

### 2.2 Role of the solvent in protein–ligand binding

Over the past decade, extensive work has focused on the modulation of the energetics of water molecules in protein binding sites by ligands. Depending on the particulars, ligand modifications can not only displace discrete ordered water molecules from a binding site, but also perturb water networks around the bound ligand or in solution, a possible source of non-additivity.^18^ A variety of free energy calculation methods have been applied to elucidate the importance of such effects in a range of binding sites. An approach that has found application in several structure-based drug design programs is the Watermap method from Friesner and co-workers.^19,20^ The technique combines clustering of explicit solvent molecular dynamics simulations with the inhomogeneous fluid solvation theory (IFST) method developed by Lazaridis to yield enthalpy and entropy estimates for discrete water molecules.^21–23^ A different, but conceptually related approach called SPAM, has been developed by Cui *et al.* and used in a range of structure-based drug design programs at GlaxoSmithKline.^24^ Because it is not clear that it is always appropriate to rationalize water properties around organic and biomolecules with a few discrete high water density sites, a discretized version of IFST that is appropriate for analyses of water behaviour in a broader context has been recently proposed by Nguyen *et al.* ^25^ Other alternatives are emerging, Gerogiokas *et al.* have developed the grid cell theory methodology to address similar issues.^26^ Careful comparative studies will be necessary to assert the merits of these different approaches. A common pitfall encountered in hydration analyses of protein binding sites is that in some cases adequate sampling of water locations can be exceedingly slow with molecular dynamics methods. The issue is typically more pronounced for simulations of holo structures that contain water-mediated interactions between protein and ligand groups. A variety of methods have been proposed to equilibrate more rapidly the water content of a binding site, these include approximate free energy methods based on double-decoupling theory,^27^ Grand Canonical Monte Carlo methods,^28^ and more rapid non-simulation based approaches based on 3D-RISM,^29^ or other energetic descriptors.^30,31^ Other open issues include, how far from a solute do energetically relevant perturbations in solvent structure extend, and how much do disordered low density hydration sites contribute to ligand binding energetics.

Molecular simulations have also been combined with biophysical measurements on a range of model protein–ligand systems to elucidate the contribution of water to binding energetics. A prominent example is the work of the Whitesides group on carbonic anhydrase.^32,33^ The picture that emerges from this body of work is that changes in water energetics upon ligand modifications contribute significantly to ligand binding energetics and detailed modelling of binding site water molecules appears necessary to reliably predict binding free energies for ligand optimisation.

Another solvent related problem that is rarely considered in current molecular simulation studies of protein–ligand interactions is that computer models typically assume the biomolecular complex is in pure aqueous solution at pH 7, and may include a discrete number of monovalent counter-ions to maintain an overall neutral net charge. The computed binding energies are then related to *in vitro* measurements that are normally performed in much more complex solutions which contain multiple other species (*e.g.* buffers, surfactants, organic co-solvents). Also, changes in ligand protonation states upon complexation are frequent. Such changes can be deduced from isothermal titration calorimetric measurements, and have potentially large effects on binding energetics.^34,35^ Mirroring accurately experimental conditions will not only require simulations of more complex mixtures, but also refined simulation protocols that enable sampling of protonation states on protein and ligand functional groups or alternative tautomeric and mesomeric forms. Several methodologies have been proposed for explicit solvent constant pH simulations of proteins in recent years.^36–39^ Refined protocols for constant pH simulations of protein–ligand complexes are a likely next milestone. A longer term horizon for molecular simulations is to elucidate the influence of cellular crowding on the energetics of protein–ligand association; little work has been done in this area owing to huge technical challenges. Some early steps have been taken by the Elcock lab that has reported in recent year simulation studies of the energetics of hydrophobic association in various salt concentrations, and crowding conditions,^40,41^ or even the stability of proteins in simplified models of bacterial cytoplasm.^42^

### 2.3 Role of conformational entropies

Another source of complexity arises from the difficulty of estimating changes in protein and ligand entropies upon binding.^43^ Brute-force direct evaluation of the absolute entropy of a system essentially amounts to sampling completely its accessible phase space, which is hardly feasible for condensed-phase simulations. Entropies of binding can be in principle indirectly obtained by van't Hoff analysis of free energies of binding computed at different temperatures. In practice, unless the simulated system is particularly simple, statistical uncertainties in the evaluated binding free energies often render the approach unreliable.

A route to protein and ligand entropies is offered by the quasi-harmonic analysis method that uses fluctuations and covariance in atomic positions described in Cartesian or internal coordinates recorded over the course of a simulation to estimate entropy through a harmonic approximation.^44,45^ This at least offers prospects for converged results, although long simulation times are required. The main drawback of the approach is that multimodal degrees of freedom (*e.g.* torsions) are not well represented by this approximation, and correlations between motions above the pairwise level are ignored. In addition, correct treatment of the diffusive behaviour of solvent molecules requires assignment of individual molecules to small spatial regions,^46^ or use of inhomogeneous fluid solvation,^21^ or cell theory approaches.^47^ The main alternative is to expand the full entropy of a system into a series of terms that account for correlations of increasing order, for instance the Mutual Information Expansion method proposed by Gilson and co-workers.^48^ Currently it appears feasible to converge low-order terms for typical drug-like small molecules. However, a similar feat for much larger protein complexes appears out of reach.^49^ Pessimistic arguments against the feasibility of converging even first order terms for proteins with molecular dynamics simulations have been put forward by Genheden and Ryde,^50^ but others have reported better success.^51^ Many efforts have been devoted to improving upon entropy expansion and quasi-harmonic approaches to estimate entropy changes upon ligand binding. Correction terms for the quasi-harmonic approximation have been proposed by Baron and co-workers.^52^ The Maximum Information Spanning Tree method proposed by Tidor and co-workers shares similarities to the Mutual Information Expansion method but exhibits improved convergence properties.^53^ Another intriguing example is provided by the Boltzmann-Quasi Harmonic method of Di Nola *et al.*,^54^ which has been recently re-evaluated by Sharp and co-workers for biomolecular complexes.^55^ The approach estimates entropy with a first order entropy expansion of internal degrees of freedoms, and pair-wise correlations with the off-diagonal terms of a covariance matrix.

Because the above methodologies make approximations, the estimated entropy contains systematic errors; such errors do not cancel out completely when comparing different ligands or when estimating contributions to entropies of binding. Consequently routine application to elucidate contribution to ligand binding energetics remains currently challenging. Robust analyses seem to require a rigid receptor approximation, or should be restricted to sets of well-defined protein conformational states for which thorough sampling of the accessible phase space can be achieved. The direct evaluation of enthalpies of binding is conceptually simpler, neglecting a pressure–volume term, one would need to evaluate differences in average potential energies for a solvated protein, a solvated ligand and a complex. However the approach also suffers from severe convergence issues which preclude routine application to biomolecular systems.

It is interesting to reflect on why it is reasonable to expect molecular simulations to compute acceptably converged free energies of binding even though entropies or enthalpies are ill-converged. The reason is that the derivation of statistical mechanics formulae to evaluate entropies and enthalpies of binding leads to expressions that involve averages over a large number of interactions between protein and solvent particles.^43^ Such terms fluctuate significantly over the course of a simulation and unreasonably long simulations are necessary to obtain sufficiently precise estimates for useful predictions. However, such slowly-converging terms cancel out exactly in expressions used to evaluate ligand binding free energies, leaving only contributions from the ligand intra and intermolecular terms, which converge much more rapidly.

### 2.4 Influence of finite-size artefacts

The problems mentioned in Section 2.3 can be largely tracked to difficulties in sampling the relevant low energy conformations of a solvated biomolecular complex. When this cannot be done reliably, repeated molecular simulations produce, sometimes qualitatively, different answers. However, in cases where reproducible predictions are possible, systematic differences with experiments are expected due to approximations in the representations of the energetics of interactions between particles. One source of error is due to the use of a finite-size box to simulate protein–ligand interactions. Finite-size artefacts are particularly apparent for simulations of charged solutes. For instance, the use of periodic-boundary conditions or cutoffs to evaluate intermolecular energetics is known to affect by several kcal mol^–1^ the free energy of hydration of simple ions.^56^ It follows that comparing computed binding affinities between ligands that differ in net charge is often problematic. Hunenberger and co-workers have devoted considerable efforts to formalize and address the technical issues. Correction terms have been derived and essentially fully account for such finite-size effects but only in the context of simple monoatomic ions.^57,58^ Recently, different correction terms have been proposed by Rocklin *et al.*,^59^ and Reif and Oostenbrink^60^ for the case of charged polyatomic particles.

### 2.5 Beyond fixed-charge classical force fields

Another major source of error includes the common representation of electrostatic fields with atomic partial charges. Multipole expansions typically reproduce better high-level quantum mechanical calculations on model compounds. A second related issue is the neglect of polarisability implied by fixed charges force fields. This likely accounts for the lack of transferability of fixed charge force field parameters, *i.e.* different parameter sets are needed to accurately model the interactions of an organic molecule in media of varying polarity, for instance water and chloroform. Several alternatives to fixed atomic partial charges force fields have been proposed.^61^ Popular models include the CHARMM Drude oscillator model where polarisability is included through addition of massless charged particles attached to the centre of an atomic particle *via* harmonic springs.^62^ A more computationally intensive alternative is the AMOEBA force field that includes induced dipoles and replaces partial charges with permanent multipoles.^63^ The QMPFF force field is also a variant that models polarisability with floating diffuse isotropic electron clouds attached to nuclei.^64^ To date, validation studies have frequently focused on modelling small molecule solvation in different media.^65^ Applications to ligand binding problems are slowly emerging, Jiao *et al.* have computed binding free energies for a small set of Trypsin ligands using the AMOEBA force field,^66,67^ whereas Khoruzhii *et al.* have used QMPFF to compute relative binding free energies for five ligands bound to three serine proteases.^68^ Much larger scale validation studies are still necessary to conclude to which extent the more elaborate treatment of electrostatic interactions featured by these force fields improves upon traditional biomolecular force fields.

A less developed alternative focuses on the coupling of classical molecular mechanical (MM) and quantum mechanical (QM) potential energy functions. Potentially this bypasses the need to derive suitable force field parameters for ligands. However because it is still very expensive to perform QM/MM simulations of biomolecular complexes, many approaches proposed so far are based on post-processing of trajectories pre-computed with a classical potential.^69,70^ Care must be taken to select a suitable level of theory to improve upon the energetics given by classical potentials. Faver *et al.* have broken down a complex of HIV-II/indinavir into 21 small fragments and computed interaction energies using a range of methodologies, comparing to benchmark calculations performed at the CCSD(T)/CBS level of theory.^71^ Semi-empirical and HF methods were in general outperformed by the empirical force field GAFF, but dispersion corrected DFT methods markedly improved results.^72^ Antony *et al.* have performed a similar study for 25 fragments of diverse protein–ligand complexes, observing also that dispersion corrected DFT results were in close agreement with benchmark calculations performed at the MP2 or LPNO-CEPA/1 levels of theory.^73^

An important current focus is on devising efficient protocols to directly sample potential energy surfaces using QM/MM representations. Many open questions remain about the best way to couple QM and MM Hamiltonians for binding free energy calculations.^74–77^ For instance, one possible strategy is to periodically accept configurations generated with an MM potential on the basis of a Metropolis test that involves evaluation of QM/MM energies.^78^ The QM region can itself be large and include several layers of solvent molecules, if linear-scaling density functional theory approaches are adopted.^79^ Understanding the influence of the size of the QM region on the computed binding energetics is also an active area of research.^80–82^

Overall it is still early days for QM/MM binding free energy calculations and further technical research is needed before routine applications in structure-based drug design can be considered.

## 3. Opportunities to help interpret biophysical measurements

### 3.1 Protein X-ray crystallography

A growing and important role for molecular simulations of protein–ligand interactions is to facilitate the connection between biophysical measurements and biomolecular structure. For instance, obtaining knowledge about the position of ordered water molecules in protein binding sites can be difficult, yet important to rationalize protein–ligand interactions. Few NMR methods can localize accurately protein hydration sites. It is also well known that the number of crystallographic waters correlates with the resolution of a protein crystal structure.^83^ In some instances, peaks in electron density in crystals initially attributed to clusters of water molecules have been later suggested to be caused by non-polar impurities.^84^ In ambiguous cases, molecular simulations can be performed to evaluate whether it is energetically unfavourable to place a water molecule at a given location.^27^ Although crystallographic structures are frequently used to rationalize binding assays performed in aqueous solutions, it is not clear whether it is appropriate to refine X-ray structures with force fields that were developed for protein simulations in aqueous conditions. Case and co-workers have been exploring this issue by performing molecular dynamics simulations of protein crystals with several different protein and water force fields.^85^ In another recent study, several microseconds molecular dynamics simulations of a peptide crystal were performed.^86^ A better agreement with the experimental data was obtaining by refining the peptide coordinates against structure factors obtained from simulated averaged electron density maps. The simulations led to the identification of additional water positions that improved model refinement. Minor populations of alternative side-chain and backbone conformations were also apparent, although this was attributed to force field errors. At present significant technical challenges must be addressed before the approach could be used more widely. In addition to the usual limitations due to force field accuracy, the water content of the unit cell often has to be deduced by running multiple simulations with a different number of water molecules until a setup that provides a unit cell size compatible with experimental data is found. Also many crystals are obtained by soaking in complex buffers and at low temperatures, correct modelling of these conditions presents additional hurdles for classical force fields. Nevertheless, there is encouraging potential for molecular simulations to contribute to the refinement of protein–ligand crystals. Particularly useful applications would focus on clarifying ambiguities in the bound conformations of ligands owing to limitations in experimental data, and identifying alternative patterns of side-chain rotamers in binding sites that may influence ligand optimization strategies.

### 3.2 Protein NMR

NMR methodologies are more commonly used routinely for ligand screening rather than structure-based ligand design efforts, in part because sample preparation and data analysis is considered more cumbersome than protein crystallography. However NMR may be the method of choice if a protein is difficult to crystallize or if protein dynamics plays an important role in modulating interactions with ligands. The field of protein NMR has a long history of using classical force fields supplemented with NMR derived restraints in simulated annealing protocols to generate structural models compatible with experimental data.^87^ A major challenge is that the experimental data available for model construction is too sparse to unambiguously define a structure, let alone a structural ensemble. Consequently explicit solvent molecular simulations with latest generation force fields are increasingly used to refine structural ensembles and back-compute experimental observables for model validation.^88^ In this area there has been renewed interest in estimating chemical shifts from structures computed from a molecular simulation to validate force fields and characterize distinct conformational states. Several empirical methods to predict backbone and methyl chemical shifts from an input structure are available for this purpose.^89,90^ There are limitations with this approach because force field errors and errors in the chemical shift predictors are intertwined. Nevertheless, the empirical approaches are currently much more efficient and also more accurate than chemical shift predictions based on quantum chemical methodologies.^91^ However quantum chemical methodologies can readily account for the presence of ligands or other molecules for which a lack of experimental data precludes the calibration of empirical descriptors.

In structure-based drug design a desirable application of protein NMR is to generate ligand binding modes based on chemical shift perturbations. Typically chemical shift perturbations are used to map binding sites onto crystal structures, but ambiguities about the details of the ligand binding mode often remain. The combination of docking methodologies with chemical shift predictions has been proposed to generate plausible binding modes in those instances.^92^ An accurate and efficient chemical shift prediction methodology by post-processing of molecular simulations with quantum chemical calculations has the potential to improve upon such protocols. Looking further ahead, the combination of chemical shift measurements with molecular simulation methods also shows promises to characterize minor conformers and for studies of flexible systems that cannot be targeted with conventional X-ray crystallography approaches, for instance intrinsically disordered proteins.^93,94^

### 3.3 Isothermal titration calorimetry

Molecular simulations also have the potential to help interpret isothermal titration calorimetry measurements of protein–ligand binding affinities. The technique is increasingly popular in drug discovery as it provides direct measurements of free energies of binding, enthalpies (and therefore entropies), stoichiometry, and changes in protonation states.^95^ However relating changes in binding enthalpies and entropies to the structural data provided by X-ray structures has generally proven very difficult. The issue is of interest because some groups have argued that measuring enthalpy changes during early-stage drug discovery activities may improve odds for clinical success.^96,97^ The essence of the argument is that ligands whose free energy of binding shows a significantly favourable enthalpic component are more likely to interact with the target through well positioned hydrogen-bonds, whereas those that bind with a significantly favourable entropic component do so by virtue of hydrophobic moieties displacing water molecules. The latter interactions are thought to increase liabilities in late stage lead optimization because they correlate with poor solubility or binding specificity.

There are reasons to doubt this interpretation. Firstly, binding entropies depend on standard state definitions, but binding enthalpies do not, thus one can change the binding enthalpy/entropy ratio simply by changing the (arbitrary) standard state definition.^15^ This makes it awkward to conclude that the binding of a ligand is dominated by an enthalpic or entropic component. Comparison of differences in enthalpic and entropic components between related ligands is however valid since these are not affected by standard state considerations. An important motivation for computing free energy components is the desire to address fundamental questions about the phenomenon of enthalpy–entropy compensation. The effect, whereby a structural modification to a ligand that decreases the enthalpy of binding is opposed by an unfavourable decrease in entropy of binding (and *vice versa*), is commonly encountered in protein–ligand complexes. However there is debate about extent, meaning and interpretation of the effect.^98^ Strong compensation between absolute enthalpies and entropies of binding have been shown to be measurement artefacts.^99^ Accounting for these errors greatly reduces the extent of entropy–enthalpy compensation in binding free energies but does not eliminate it completely.^100^ Gilson and co-workers have also reported an elegant computational study of the relative enthalpic and entropic components of different conformational states of the protein BPTI.^101^ Major findings from this study are that distinct protein conformational states that marginally differ in relative free energy can have much greater differences in their enthalpic or entropic components. This has major implications for the feasibility of enthalpic optimization of ligands, for instance a ligand that intrinsically binds to a protein by decreasing the enthalpy of the system may actually appears to bind with a large entropy increase if it preferentially stabilizes a protein conformation that has greater entropy than the dominant apo conformation. Such effects may well contribute to apparent entropy–enthalpy compensation and complicate the interpretation of calorimetric measurements of entropies and enthalpies to guide lead optimization.

## 4. Opportunities to suggest new ligand design strategies

Molecular simulations provide enormous details in the molecular interactions that underpin protein–ligand association. Although routine high-throughput applications are not widespread, simulations have a long history of suggesting strategies for optimizing ligand affinities.

### 4.1 Modulating the stability of binding site water molecules

A frequent dilemma molecular modellers face during ligand optimization is whether or not to attempt to displace ordered water molecules that are apparent in the crystallographic structure of a complex with a hit compound. The rationale goes back to Dunitz who reasoned that, owing to steric restraints, there must be fewer ways for a water molecule to form hydrogen-bonds with protein–ligand donor–acceptor groups. Since motions are hindered, the entropy of water in a typical binding site should lie somewhere between the entropy of liquid water and ice.^102^ Consequently, if it is feasible to prepare an analogue that incorporates a moiety to displace a bound water molecule and establish hydrogen-bonding interaction similar to that of the displaced water molecule, the free energy of binding of the ligand will benefit from an entropic gain due to release of the water molecule in bulk. In practice achieving this outcome is difficult because accurate positioning of the water displacing moiety may not be feasible, and any mismatch in hydrogen-bonding interactions will rapidly offset an entropic gain due to water displacement. Molecular simulations can capture such effects, and have been shown to be useful to rationalize when an ordered water molecule can be productively displaced.^103^ There is however debate about the magnitude of the entropy gain that could be achieved by displacing a water molecule. The debate partly arises because formally it isn't possible to unambiguously partition the free energy into contributions from individual water molecules. Simulation methodologies that estimate the free energy of a water molecule by partitioning the average potential energy of the system and by estimating solvent entropy by various means often assign unfavourable free energies with respect to bulk for some water molecules at biomolecular interfaces. Though such water molecules are deemed locally “unstable”, their presence is necessary to stabilize interactions between neighbouring particles.^104^ Additionally, detailed experimental and computational studies have revealed different plausible scenarios for water stability at biomolecular surfaces, for instance water molecules near hydrophobic surfaces may be entropically favoured and enthalpically disfavoured compared to bulk conditions. Complex changes in thermodynamic signature have been inferred from careful calorimetric studies of a series of analogues binding to thermolysin by Klebe and co-workers and Whitesides and co-workers.^33,105^ Overall, the conclusion is that water molecules can be productively displaced by improving the enthalpy or entropy of binding. Detailed knowledge of binding site hydration at a level currently afforded by a molecular simulation appears necessary for predictive applications.

### 4.2 Modulating conformational flexibility

Another simulation-inspired strategy that has been less frequently attempted is to modify ligands to increase structural flexibility of the target protein. The expectation is that this will favourably increase protein entropy, but inevitable associated changes in solvent entropy complicate the picture. The strategy may also appear counter-intuitive, high affinity ligands are typically obtained by tightening interactions with the receptor. However Nature provides examples, for instance the N-terminal domain of the protein MDM2 contains a partially disordered lid that has been shown to exist in equilibrium between open and closed conformations, the latter covering a binding site for partner protein p53.^106^ Peptidic ligands derived from p53 shift the equilibrium to an open and more flexible lid conformation, which is expected to partly offset the entropic cost for structuring of p53 peptides in the MDM2 binding site.^107^ In the realm of small molecule ligands, NMR has long shown for instance that ligand binding to the protein DHFR modulates protein flexibility in non-trivial ways, with some regions becoming more flexible upon ligand binding.^108^ Another similar example is provided by saccharides binding to the carbohydrate recognition domain of Galectin-3.^109^

A rationale strategy has been explored in the context of kinase inhibition by Crespo and Fernandez.^110^ Substitution of a hydrogen atom by a chloride atom on the pyrimidine ring of the drug imatinib was proposed to promote an unfavourable interaction with the activation loop of the D861V C-kit kinase mutant, that binds this drug poorly. The strategy was to improve affinity by increasing disorder in the activation loop in the complex. The feasibility of the approach was supported by molecular dynamics simulations and free energy calculations. The ligand was synthesized and activity measured with a spectrophotometric assay. Gratifyingly, although imatininib binds poorly C-kit D861V, the analogue was about 250 times more potent. This suggests the strategy is viable, although more work is required to confirm that the origin of affinity improvement is indeed increased protein disorder. Beyond this example, limited evidence has been accumulated so far to suggest how widely applicable the strategy is, and whether the practice would produce high-quality leads.

By contrast similar strategies that focus on the ligands themselves have long been pursued by molecular modellers. The concept is that most drug-like small molecules contain a number of rotatable bonds and often adopt multiple diverse low-energy conformations in solution. This is generally expected to disfavour binding since owing to steric constraints, much fewer conformations are assumed to be available for a ligand in a protein binding site. Consequently structural modifications that rigidify a ligand in solution should decrease this entropic contribution to the free energy of binding. Recent work from Jorgensen and co-workers suggest that this conformer “focusing” effect,^111^ may explain at least in part the “magic methyl” effect,^112^ whereby in some instances addition of a methyl group to a ligand to fill available space in a binding site improves binding affinity *ca.* 100 fold, much more than typically observed. In the cases investigated, the effect was more pronounced when a methyl group substituted a hydrogen atom on *ortho* positions of aryls.

Although the concept is sound, systematic application presents pitfalls. Counter-intuitive results have been reported for peptide analogues binding to the Grb2 SH2 domain. To improve the binding affinity of a parent series, a cyclisation strategy was pursued by introduction of a cyclopropane ring in the peptide backbone. The intention was to thus preorganise a phosphotyrosine side-chain in its bioactive conformation. Unexpectedly, the resulting analogues were however shown to bind with a greater entropic penalty than the parent compounds.^113^ Crystal structure analyses of constrained and unconstrained ligands in complex with the target did not reveal significant differences in binding modes. Detailed molecular simulation studies were pursued by Shi *et al.* to rationalize these results.^114^ The major findings were that the experimental results were qualitatively reproduced by the simulations. Analysis of the computed conformational ensembles of the bound and unbound ligands revealed that although the cyclopropane ring modification does indeed locally constrain the bound ligands, it also hinders formation of intramolecular electrostatic interactions between surrounding side-chains when the ligands are unbound in solution. Consequently, the constrained ligands in solution adopt more flexible, extended conformations, and lose more entropy upon binding. Overall it thus appears that the detailed understanding of the conformational flexibility of ligands bound to their target protein and also in solution afforded by molecular simulation provides a way to test optimization hypotheses based on conformational constraints.

### 4.3 Predicting alternative receptor conformations for virtual screens

Another emerging powerful role for molecular simulations is to suggest plausible alternative protein conformational states for docking calculations.^115^ Since proteins are generally flexible macromolecules, conformations that differ from an experimentally derived structure may be adopted in solution with a negligible or small energetic cost. Such alternative conformational states may be more attractive than those apparent in the starting structure from a small molecule drug design standpoint. The approach has been coined “ensemble-based drug design” by several groups to highlight the focus on the analysis of computer generated structural ensembles of a biomolecule. The popularity of the approach is increasing because for several proteins, molecular dynamics simulations of apo structures on a *ca.* ∼10–1000 ns timescale reveal conformational fluctuations that significantly modify small molecule binding sites or even reveal completely new cavities. In some instances, it has been possible to retrospectively dock known ligands that would otherwise not fit into snapshots sampled from apo structures simulated with such protocols. Early work in this area was performed by McCammon and co-workers, simulation studies on the flexibility of the protein HIV-integrase led to the characterization of a cryptic alternative binding site conformation that could accommodate suitably analogues of known ligands.^116^ These findings impacted on then on-going optimization of HIV-integrase inhibitors at Merck.^117^ More recent work on the tumour suppressor p53 by Amaro and co-workers illustrates a possible drug design workflow.^118^ Mutations that destabilize the DNA binding domain of p53 are frequently observed in cancers, Fersht and co-workers have shown that small molecules can rescue p53 function by binding to and stabilizing the p53 DNA binding domain.^119^ Molecular simulations of the wild-type p53 DNA binding domain and several mutants were performed by Wassman *et al.* ^118^ Ensemble-analyses indicated frequent formation in wild type and several mutants of a transient cavity that exposed Cys 124 to the solvent. This observation was of importance since prior biochemical evidence by Fersht and co-workers suggested that such cysteine may be a covalent site for known alkylating agents stabilizers of p53.^120^ Docking of some of these agents into the computed pocket produced reasonable binding modes to enable alkylation of the sulfhydryl group of Cys 124. Next, no p53 stabilisation was apparent when the alkylating agent PRIMA-1 was experimentally tested against a C124A p53 mutant. Subsequently virtual screens by docking against a panel of conformations of the transient cavity sampled during the simulations were performed. The exercise ultimately led to the selection of 45 compounds for assays. One of them, stictic acid, was shown to increase p53 levels in a cancer cell line at micromolar concentrations.^118^ Further work is needed to confirm the predicted binding modes, mechanism of inhibition, and to optimize the hit into a viable lead. Nevertheless, the strategy illustrates well current capabilities and limitations.

Extensive work is needed for ensemble-based approaches to become a routine hit finding strategy. It is unclear to which extent in general conformations computed from a molecular simulation of an apo protein will adopt holo-like conformations. The approach appears most suited for targets that recognize ligands through a conformational selection mechanism, and small associated conformational changes. If molecular recognition operates instead by a primarily induced-fit mechanism, then the computed structural ensemble may add little additional value to the starting crystal structure. This is because it is difficult to perform accurate high-throughput docking screens whilst enabling protein flexibility. It may also be quite challenging and time-consuming to narrow a set of ten to a hundred thousand snapshots to a selected few structures that are most suitable for follow-up virtual screens. Protein ligandability scoring functions may be used to evaluate the potential of each computed transient cavity to bind a ligand.^121^ This should also be done in conjunction with estimates of the stability of the computed cavities; presumably in most instances there is an unfavourable energetic cost associated with formation of the transient cavity. This energetic cost must be offset by ligands binding to a transient conformation. Where possible, experimental evidence that the computed conformation is plausible should also be sought to justify follow-up work at different steps of the workflow; a worst case scenario is that much efforts are devoted to chasing cavities that do not exist in reality.

### 4.4 Predicting allosteric interactions

A practical issue rapidly encountered upon analysis of computed protein structural ensemble is that some cavities deemed attractive for binding ligands may not overlap with the substrate/native partner binding site. In those cases additional evidence that ligand binding to such pockets will lead to modulation of biological function is desirable. The proposal is particularly attractive in those cases where the native binding site may be deemed too difficult to target with a small molecule, or offers little prospects for achieving sufficient binding selectivity. Several computational methodologies have been proposed to infer allosteric coupling between sites, but obtaining a clear yes/no answer from these tools is currently sufficiently difficult that it is wise to obtain corroborating experimental data before following up with a virtual screen. Popular non simulation methods include Gaussian Network Models that can rapidly suggest allosteric pathways, typically from analyses of contacts between C_α_ atoms.^122^ The method appears to capture reasonably well some putative allosteric pathways but is quite sensitive to small atomic displacements. Analyses are however sufficiently rapid for post-processing structural ensembles computed by molecular simulations. Much more expensive simulation-based approaches rely on information-theoretic analyses of perturbations in probability distributions functions of protein degrees of freedoms, typically torsions.^123,124^ The simulation approach is in principle more sensitive to subtle allosteric mechanisms that could involve for instance side-chain flips. However a major issue lies in achieving a significant signal-to-noise ratio; it is currently difficult to obtain reproducible results for the same reasons that it is difficult to obtain converged protein entropies. A current focus for the field is to improve the reproducibility of simulation based predictions of allosteric interactions.^125^ Given these caveats, routine reliable *a priori* prediction of allosteric sites in the absence of experimental data to support simulation based hypotheses still appears some years into the future.

Overall ensemble-based drug design techniques offer exciting prospects to deliver small molecule ligands in new ways. The current applications focus largely on generating alternative conformational states from existing crystallographic data and on inferring allostery from analyses of structural ensembles. Looking ahead, additional biomolecular recognition processes such as protein aggregation could in principle be tackled with similar methodologies; one could for instance seek plausible binding sites that would stabilize a protein in a monomeric form and disfavour oligomerisation.^126^

## 5. Opportunities to routinely optimize ligands

### 5.1 Interplay with other computational approaches

A long stated goal of biomolecular simulations is pervasive application in structure-based hit-to-lead and lead optimization problems. It is useful to clarify contexts where alternatives to molecular simulations may be currently more cost effective. Firstly, in instances where high resolution structural data are limited, the methodology is not easily applicable. Also, the range of structural rearrangements that can be routinely simulated over the course of a reasonably expensive molecular simulation is limited. “Double blind” modelling studies, whereby the structure of a putative ligand is rapidly docked into a low quality homology model of the target protein will often lead to the pursuit of qualitatively incorrect hypotheses. Secondly, projects that focus on generating novel ligands similar to existing ones may in some instances reach the desired objectives more efficiently through ligand-based and chemoinformatics methodologies. This is especially true for targets where extensive structure–activity relationships datasets are available, *e.g.* kinases, certain families of GPCRs.^127^ Lastly, this is not always obvious to collaborators, projects with no known ligands and that lack structural data on the target offer little prospects for rapid insights from a molecular simulation.

Today short molecular dynamics simulations of protein–ligand complexes are frequently used by modellers once a hit structure has been identified after an initial experimental or computational library screen, but before structural data to confirm the interactions have become available. Emphasis is on qualitative analyses to explore for instance hypotheses about putative binding modes, or to propose analogues to generate structure–activity relationships. Quantitative predictions of free energies of binding remain difficult for the reasons outlined earlier, but there are reasons to expect that scoring methodologies based on molecular simulations should perform better than empirical scoring functions. Through careful analyses of errors in models of protein–ligand energetics, Merz and co-workers have concluded that the averaging of interaction energies over many conformations, which is implicit in molecular simulations, reduces systematic errors compared to approaches based on single-point energetics.^128^ Simulation based scoring methods such as Molecular Mechanics–Poisson Boltzmann Surface Area have been extensively explored over the past 10–15 years, but the performance appears to be too much system dependent for routine applications in ligand optimisation.^129^ Today there is much interest in using molecular simulation protocols that predict relative free energies of binding using methodologies like free energy perturbation or thermodynamic integration. This is partly because the approaches rely on firm theoretical grounds and partly because a few laboratories have led the way and applied extensively such methodologies; a notable example is the work of Jorgensen and co-workers on HIV reverse transcriptase and other targets.^130^

### 5.2 Automation is a growing concern

Since approximate predictions are expected in practice due to incomplete configurational sampling and inexact potential energy functions, benefits are expected to be more apparent when predictions are performed on a large number of ligands. Nowadays securing sufficient computing time to perform such calculations on datasets of 10–1000 ligands is not a major bottleneck. Organizations that do not maintain in house computing clusters could periodically secure such resources through cloud computing infrastructures. However a major practical issue for large scale applications is the need to correctly prepare input files and analyse the output of the simulations. There has traditionally been little concern on this in academia where many of the software packages that implement alchemical free energy calculations originate. The setup and analysis of a free energy calculation is probably about one order of magnitude more complex than a typical docking calculation. Automation of binding free energy calculations would bring several benefits to the field. Firstly, it will facilitate large scale retrospective studies of the accuracy and precision of different protocols; a common criticism is that free energy calculation methodologies are not validated on sufficiently large datasets to truly assess predictive power. Secondly, it will facilitate reproducibility of simulation studies; the setup of input files is frequently insufficiently documented in publications to make it difficult to reproduce published results. Thirdly, it will facilitate wider adoption by time-pressed molecular modellers and other biomolecular scientists. Achieving these goals will not be easy because there is limited consensus between experts on the most robust protocols to adopt. Workflows for high throughput studies should be designed to be flexible and extensible, best practices will keep evolving. Several workflows that automate diverse aspects of binding free energy calculations have been recently reported.^131–133^

### 5.3 Plausible applications

The final goal is to aid rather than automate drug design, and many scenarios for the most effective integration of simulations with experiments ought to be further explored. Molecular simulation protocols that predict free energies of binding would not be used to screen millions of compounds for some time, but could conceivably be used as a post virtual screen filter for perhaps 10^2^–10^3^ compounds. As this is a rather small number, additional filters should be applied prior to performing free energy rescoring – perhaps putting emphasis on chemical diversity. In addition, the structural diversity of compounds pulled from a large library may make it more challenging to obtain converged estimates of binding affinities with typical free energy calculation methods. Fragment-based screens offer higher compatibility with molecular simulations. Firstly, the fragment libraries screened are typically smaller, *ca.* 10^2^–10^4^ compounds, so a greater fraction could be immediately screened using free energy methods. Secondly, the fragments typically contain fewer rotatable bonds and it should be less challenging to obtain converged free energies of binding. If there is ambiguity, free energy calculation protocols can also be used to rank-order putative binding modes of the same ligand.^134,135^ Thirdly, fragment-based drug design approaches frequently rely on structural and biophysical and data to characterize hits,^136^ which is important for robust application of molecular simulations. For hit optimization, the practical utility of molecular simulations also depends on the available resources for analogues synthesis. As with other ligand optimization techniques, the input of molecular simulations is greater if the potential for diverse structural optimization strategies is assessed early. This helps prioritize the selection of suitable synthetic strategies for preparation of focused analogues libraries.

Looking ahead, if reasonable success in optimizing hits is demonstrated for a broad range of targets, there is prospect that molecular simulations will more frequently tackle other challenging ligand optimization problems. For instance, permeability may be tuned with simulations of diffusion across models of biological membranes;^137^ plasma binding levels may be optimized through simulations of compounds bound to Human Serum Albumin;^138^ metabolic stability controlled through simulations of various Cytochrome P450 complexes;^139^ binding selectivity to disordered proteins could be adjusted,^140^ and so on. Major challenges here involve developing reliable protocols to quantify ligand interactions with promiscuous targets; in many cases the ligands likely adopt multiple binding modes or occupy multiple distinct sites.

## 6. Conclusions

Molecular simulations provide truly outstanding opportunities to exploit molecular recognition principles that are very difficult to observe with experimental techniques. The continuing trends in high-performance computing suggest that molecular simulations are poised to take on wider significance in structure-based drug design in the coming years. In spite of the hopes, it is prudent to acknowledge that the concept of using computers to optimize ligand interactions with the aid of structural data is not new and the field of computer-aided drug design has promised fast and accurate predictions of binding affinities for decades. It should be well appreciated that, given the complexity of biomolecular interactions, routine reliable predictions of binding affinities from molecular simulations is truly a Holy Grail for computational chemistry. Steady incremental progress towards this goal is more realistic. Extensive retrospective and prospective methodological studies on substantial datasets will be instrumental to diagnose and address shortcomings in sampling algorithms and energy functions. The major goal is to demonstrate more accurate predictions but emphasis should be put on reproducibility and statistical significance. Efforts should also be devoted to defining robust, partly automated, simulation protocols for classes of ligand-binding problems that emerge as the most tractable. The goal should be to facilitate rapid large scale studies by non-technical experts. This will naturally lead to the testing of new scenarios for coupling molecular simulations and experiments to tackle increasingly complex drug design challenges.

## Conflicts of interest

The author declares no competing financial interests.

## Funding sources

JM is supported by a University Research Fellowship from the Royal Society. The research leading to these results has received funding from the European Research Council under the European Union's Seventh Framework Programme (FP7/2007-2013)/ERC grant agreement no. 336289.
